# Repellent efficacy of a new combination of fipronil and permethrin against *Lutzomyia longipalpis*

**DOI:** 10.1186/s13071-018-2831-7

**Published:** 2018-04-16

**Authors:** André Antonio Cutolo, Fredy Galvis-Ovallos, Elisangela de Souza Neves, Fabiano O. Silva, S. Theodore Chester, Becky Fankhauser

**Affiliations:** 1 Boehringer-Ingelheim Saúde Animal, Fazenda S. Francisco s/n°, Paulínia, (SP) 13140-970 Brazil; 2 Universidade de São Paulo, Faculdade de Saúde Pública, Av. Dr Arnaldo, 715, São Paulo, (SP) 01246-904 Brazil; 3 Nowavet, Avenida Ernesto Lopes, 104B, Centro, Coimbra, (MG) 36550-000 Brazil; 4 Boehringer Ingelheim, 3239 Satellite Blvd., Duluth, GA 30096 USA

**Keywords:** Repellent, *Lutzomyia longipalpis*, Sand flies, Fipronil, Permethrin, Leishmaniosis

## Abstract

**Background:**

*Lutzomyia longipalpis* is the main vector of *Leishmania infantum*, the agent of canine and human visceral leishmaniosis in the Americas. Considering that the dog is the main domestic host of the parasite, repellent treatment is a measure that might contribute to the prevention of canine visceral leishmaniosis. The repellent efficacy of a single treatment of a new spot-on topical combination of fipronil and permethrin (Frontline Tri-Act®, Merial, now part of Boehringer-Ingelheim) to repel *Lu. longipalpis* sand flies was evaluated.

**Methods:**

Sixteen healthy Beagle dogs, eight females and eight males, weighing 8.4–14.4 kg, and 2 to 4 years-old were included in the study. Animals were blocked on decreasing body weight and randomly allocated within the blocks to one of two treatment groups of eight animals each. Dogs in Group 1 were untreated and Dogs in Group 2 were treated with a combination containing 67.6 mg/ml fipronil + 504.8 mg/ml permethrin (Frontline Tri-Act®) once on Day 0. Sand fly exposures were performed on Days 1, 14, 21 and 30 with *Lu. longipalpis* female sand flies. After 65 (± 15 min), sand flies were assessed for engorgement status.

**Results:**

The percent repellency of the treated group compared to the untreated control group was 95.7, 94.3, 81.7 and 72.2% for exposure days 1, 14, 21 and 30, respectively. The two treatment groups were significantly different for all exposure days (*P* ≤ 0.016 for days 1, 14, 21 and 30). No adverse reactions were observed during the study.

**Conclusion:**

A single topical administration of a new combination of fipronil and permethrin demonstrated a significant repellent effect against *Lu. longipalpis* bites as soon as it was applied on the dogs and its repellent efficacy lasted for 4 weeks with results greater than 80% for 3 weeks. The results suggest that in endemic areas the regular application of the new combination could contribute to protect dogs from *Leishmania* infection and therefore serve as an additional tool for the prevention of canine visceral leishmaniosis.

## Background

Visceral leishmaniosis (VL) is a zoonotic protozoan vector-borne disease that affects humans, canine and other mammalian species. In the Neotropics its etiological agent is *Leishmania infantum*. The disease is potentially fatal if untreated and is endemic in Brazil, Argentina and Paraguay, where the number of cases is increasing and its geographical range is expanding [1–4]. VL also occurs in Mexico, Colombia, Venezuela, Costa Rica, Guatemala, Honduras, Nicaragua, Bolivia and Guyana [5]. A total of 48,720 human VL cases have been reported in the Americas from 2001 to 2014 with an annual mean of 3480 cases. Most of these cases (46,976; 96.42%) have been reported in Brazil including 3690 human deaths from 2000 to 2015 [5].

*Leishmania infantum* parasites are transmitted by phlebotomine sand flies. In the New World the main vector is *Lutzomyia longipalpis*, a species adapted to sylvatic and rural [6], urban and peri-urban environments [7]. This vector species occurs from Mexico to Argentina [8] and is progressively expanding its distributional range in Brazil, Paraguay, Argentina [3] and Uruguay [4], and consequently expanding the risk for human and canine VL cases in these areas [8].

The dog is the most important domestic reservoir of *Leishmania infantum* [8]. Female sand flies may become infected after feeding on infected dogs and may then transmit the promastigote parasite to other mammals (mainly dogs and humans) through subsequent bites [9]. One potential control measure to break the transmission cycle of VL is the prevention of *Leishmania* infection in dogs [10]. The use of topical insecticides in dogs has been shown to be efficacious in preventing blood-feeding of phlebotomine sand flies [11–14].

This paper reports the results of a study conducted in Brazil assessing the repellent efficacy of a new spot-on topical combination of fipronil and permethrin (Frontline Tri-Act®, Merial, now part of Boehringer-Ingelheim) against the main vector of human and canine VL in the Americas, *Lu. longipalpis*. This combination is intended to provide both repellent and insecticidal-acaricidal effects against several ectoparasites of dogs such as fleas (*Ctenocephalides felis* and *C. canis*), ticks (*Ixodes ricinus*, *Rhipicephalus sanguineus* and *Dermacentor reticulatus*), sand flies (*Phlebotomus perniciosus*), stable flies (*Stomoxys calcitrans*) and mosquitoes (*Aedes albopictus*, *Aedes aegypti* and *Culex pipiens*) [12, 15–20].

## Methods

The study was conducted according to Good Clinical Practices (GCP) as described in the International Cooperation on Harmonisation of Technical Requirements for Registration of Veterinary Medicinal Products (VICH) guideline 9 [21].

### Animals

Adult Beagle dogs were used in the study and had not been exposed to ectoparasiticides for at least three months prior to enrollment in the study. Eight males and eight females, weighing 8.4–14.4 kg, two- to four-years-old, were included in the study. The dogs were housed in groups until Day -1 (i.e. until the day immediately preceding the start of the experiment) and then individually until the end of the study. No concurrent medication, except for the anesthetics used during the sand fly exposures were given during the study. The dogs were managed similarly and with due regard for their well-being. Animals were handled in compliance with Nowavet and Merial Animal Care and Use Guidelines, and in compliance with the Brazilian regulatory requirements. The dogs were acclimated to the study conditions prior to treatment and were observed at least once a day for general health conditions throughout the study. No susceptibility test to sand flies was performed pre-treatment so it was assumed dogs which were from the same breed were equally susceptible.

### Allocation and treatment

To allocate the dogs, blocks were formed based on decreasing body weight, with the two dogs with the highest body weight forming block 1, the next two forming block 2, and so on, until all dogs were assigned to a block. Within blocks, each dog was randomly allocated to one of the two treatment groups. Six additional dogs (three treated and three untreated) were kept as spare animals. Dogs in Group 1 served as untreated control animals. Dogs in Group 2 were treated on Day 0 with the topical combination of permethrin and fipronil, at the recommended commercial dose (1.0 ml for dogs < 10.0 kg, and 2.0 ml for dogs > 10.0 to 20 kg, delivering a minimum dose of 6.76 mg/kg fipronil and 50.48 mg/kg permethrin). The dose was applied by parting the hair and applying the formulation directly onto the skin on the dorsal midline of the neck. The total volume was divided into two approximately equal portions. One fraction was applied between the base of the skull and the shoulder blades and the other fraction was applied at the front of the shoulder blades. The dogs were observed prior to treatment and hourly for approximately 4 h following treatment administration.

### Sand fly exposures

*Lutzomyia longipalpis* sand flies used in the study were laboratory-reared and came from a colony located at Departamento de Parasitologia, Instituto de Ciências Biológicas (ICB), Universidade Federal de Belo Horizonte (UFMG), Belo Horizonte, MG, Brazil. The colony has been active for approximately five years and the original parental insects were obtained from the city of Teresina, Piauí State, Brazil.

Sand fly exposures were performed on Days 1, 14, 21 and 30. Prior to each exposure, animals were anesthetized with an intramuscular combination of acepromazine (0.1 mg/kg), ketamine (11 mg/kg) and midazolam (0.5 mg/kg), or ketamine (22 mg/kg) and xylazine (1.1 mg/kg) and placed individually into sand fly proof exposure cages. Exposures on control and treated dogs were performed in separate areas of the building and handled by different personnel to avoid potential contamination. An average of 79.0 three-to-five-day-old female phlebotomine sand flies were released into the exposure cages, per dog, per exposure, together with an average of 58.2 males to stimulate biting and landing behavior. Exposures were done during the night period in total darkness in order to maximize the natural nocturnal biting behavior of phlebotomine sand flies. After 65 (± 15) min, all sand flies (live and dead) were recovered and placed in a container, then counted and categorized as engorged or non-engorged.

### Data analysis

Percent sand fly repellency was calculated using the proportion of engorged (alive + dead) sand flies at the end of each post-treatment exposure period transforming to the natural logarithm of (count + 1) for calculation of geometric means (GM) of the proportions by treatment group. Percent repellency was expressed as the percent reduction in fed sand flies of the treated group compared to the control group at each post-treatment exposure day: 100 × [(C-T)/C], where C is the GM of the control group and T is the GM of the treated group. The individual dogs’ proportions of engorged sand flies from the two groups were compared using the nonparametric aligned rank test [22] that adjusted for the body-weight-blocks. SAS Version 9.4 was used for all testing, which was two-sided and used a significance level of 5%.

## Results

No health abnormalities were observed on the animals after treatment during the whole period of the study, including observations conducted for 4 h immediately after treatment. One dog presented intolerance to anesthesia during exposure to phlebotomine sand flies on Day 1. The exposure on Day 1 was completed but the dog was then removed from the study and replaced by one of the spare animals from the same treatment group, same gender, same breed and similar weight for the following exposures (Days 14, 21 and 30).

Untreated control dogs had high numbers of engorged sand flies at the end of the exposure period at all time points, with engorgement percentage means among the total exposed females of 84.6, 98.8, 98.7 and 99.4% for Days 1, 14, 21 and 30, respectively (Table 1). This feeding behaviour on control dogs shows a robust and aggressive phlebotomine sand fly strain population.

**Table 1:** Individual engorged-to-total^a^ count ratios of *Lutzomyia longipalpis* female phlebotomine sand flies per dog per exposure day with respective percentage of engorgement

		Day 1		Day 14		Day 21		Day 30	
Group 1	Dog ID	Ratio	%	Ratio	%	Ratio	%	Ratio	%
T1	206	91/94	96.8	105/105	100	60/63	95.2	126/126	100
T1	292	63/117	53.8	126/126	100	61/62	98.4	108/108	100
T1	252	68/68	100	99/106	93.4	53/53	100	125/126	99.2
T1	290	69/69	100	97/97	100	61/61	100	109/110	99.1
T1	204	67/84	79.7	136/136	100	69/69	100	66/68	97.1
T1	251	53/61	86.9	95/95	100	46/46	100	82/82	100
T1	235	40/52	76.9	73/75	97.3	65/65	100	89/89	100
T1	211	63/70	90.0	136/137	99.3	68/71	95.8	100/100	100
T2	291	3/34	8.8	7/73	9.6	16/72	22.2	22/71	30.9
T2	241	1/48	2.0	7/112	6.2	10/45	22.2	21/101	20.8
T2	232^b^	2/71	2.8	–	–	–	–	–	–
T2	237	1/39	2.5	6/76	5.2	15/57	26.3	20/60	33.3
T2	213	3/46	6.5	5/89	5.6	6/39	15.4	44/124	35.5
T2	254	1/62	1.6	3/83	3.6	8/55	14.5	10/77	12.9
T2	203	6/65	9.2	8/89	8.9	7/28	25.0	15/105	14.2
T2	209	1/74	1.3	3/102	2.9	4/71	5.6	44/103	42.7
T2	220^b^	–	–	4/73	5.5	12/48	25.0	39/72	54.1

^a^Ratio of engorged sand flies over total sand flies used for exposure (live plus dead insects)

^b^Animal no. 232 was removed from the study after Day 1 and replaced with animal no. 220

*Abbreviations*: T1, untreated control; T2, Frontline Tri-Act®/Frontect®

The percent repellency of the treated group compared to the untreated control group was 95.7, 94.3, 81.7 and 72.2% for exposure Days 1, 14, 21 and 30, respectively. The two treatment groups were significantly different for all exposure days (*χ*^2^ ≥ 5.8454, *df=*1, *P ≤* 0.016) (Table 2).

**Table 2:** Proportion of engorged *Lutzomyia longipalpis* female sand flies (geometric mean) in treated and untreated groups according to exposure day

Exposure day	Untreated group	Treated group	Repellency (%)	*P*-value^a^
1	84.1	3.6	95.7	0.014
14	98.7	5.6	94.3	0.016
21	98.7	18.0	81.7	0.016
30	99.4	27.6	72.2	0.016

^a^Proportions compared through nonparametric aligned rank test, two-sided, significance level of 5%

## Discussion

The results of this study demonstrate the high repellent effect of the new spot-on topical combination of fipronil and permethrin (Frontline Tri-Act®) against the sand fly *Lu. longipalpis*. Previous studies demonstrated that the topical combination of permethrin and fipronil provides repellency against several species of insects and ticks in dogs. Regarding the mosquito species of the Culicidae Meigen, 1818, repellency against *Ae*. *albopictus* was ≥ 93.4% through Day 21 and 86.9% on Day 28; for *Ae*. *aegypti* it was ≥ 91.0% through Day 35, and for *Cu*. *pipiens*, repellency was ≥ 90.4% through Day 28 [18]. Excellent repellency was also demonstrated against *St*. *calcitrans* stable flies with ≥ 96.6% repellency through Day 28, and 88.7% on Day 35 [19]. The product also showed good results *versus Ph*. *perniciosus*, an important vector of VL in the Old World with repellency results of 98.2, 98.5, 99.2, 90.9 and 90.3%, for Days 1, 7, 14, 21, and 29, respectively, post-treatment [12].

Topical application of pyrethroid-based insecticides to dogs in aqueous baths [23] or top spot pour-on formulations [13, 24–27] and impregnated collars [28, 29] have been described and have been shown to protect dogs against bites of phlebotomine sand flies and/or to demonstrate a prevention of transmission effect on arthropod borne agents, including leishmaniosis in the Old World. Molina et al. [24] evaluated the efficacy of a 65% topical permethrin formulation on two dogs challenged with *Ph. perniciosus* on days -8, 0, 7, 14, 21, 28, 35, 49, 70 and 91 and observed an anti-feeding effect higher than 90% until day 28. On day 35 the efficacy was 79.8%. However, there are few studies reported in peer reviewed journals evaluating the repellent and/or insecticidal efficacy of commercial veterinary products *versus* New World’s phlebotomine sand flies. Anti-feeding efficacy has been evaluated *versus* the main vector of canine visceral leishmaniosis, *Lu. longipalpis* [29] and also for vector species of cutaneous leishmaniosis (CL) like *Migonemyia migonei* [29] and *Nyssomyia intermedia* (*sensu lato*) [30].

David et al. [31] reported good results on the anti-feeding efficacy of a deltamethrin 4% impregnated collar on four treated *versus* three untreated dogs exposed for two hours to colony-reared *Lu. longipalpis* and *Mi. migonei* sand flies. Exposures to sand flies were performed on weeks 3–4, 7–8, 11–12, 15–16, 22, 26–27 and 35–36 with anti-feeding efficacy results against *Lu. longipalpis* of 99.3, 100, 100, 96, 96, 90 and 96%, respectively, and 98.3, 100, 100, 99.3, 94.3, 91.8%, respectively, for *Mi. migonei*.

Percentage of engorgement of female sand flies that fed on untreated dogs, considering all exposures during the study were 83% (1737/2,094) for *Lu. longipalpis* and 76% (1555/2035) for *Mi. migonei*.

Reithinger et al. [30] tested different commercial products *versus Ny. intermedia* (*s.l.*)*,* a vector of *Leishmania braziliensis* in dogs, in a rural area of southern Brazil in which CL is endemic [30]. On Day 0, five dogs received deltamethrin (DM) 4% impregnated collars, three dogs received diazinon (DZ) 15% impregnated collars, three dogs were treated with 65% permethrin (P) pour-on, three dogs with 15% fenthion (F) topical formulations, and three dogs were untreated controls. Non-anesthetized dogs were exposed overnight to wild caught sand flies on three different occasions [5 to 12, 32 to 36 and 58 to 65 days post-treatment (PT)]. Percent reduction in sand fly blood-feeding for the three exposures was 44.1, 31.6 and 49.2% for P; 37.6, 56.7 and 68.5% for DM; 4.2, 41.4 and 43.1% for F; and 37, 1.4 and 3.4% for DZ. In the absence of treatment, the mean engorgement rate of the female sand flies was 42%. During the first exposure, the most effective product was topical permethrin reaching only 44.1% efficacy, however there was no significant difference at the 5% significance level between the number of blood-fed sand flies from treated *versus* non-treated dogs. The deltamethrin collar had the highest efficacy overall, reaching maximum anti-feeding efficacy of 68.5% around 60 days PT.

Pour-on formulations containing pyrethroids have an important benefit since an immediate repellent effect is obtained, thus protecting the animal with the maximum efficacy within a few hours after treatment as shown in the present study. Results of the present study show that by 24 h post-treatment an efficacy of 95.7% is obtained. This is in contrast to deltamethrin-impregnated collars [30] for which the maximum repellent efficacy occurs two to three weeks after initial treatment. Since potential protection against sand fly transmitted diseases to individual dogs depends solely on their anti-feeding effect, adequate repellent efficacy is mandatory for the prevention of *Leishmania* infection in susceptible dogs. This new combination product showed rapid onset of effect with maximum repellent action within 24 h after treatment.

It is important to highlight that the anti-feeding efficacy results observed in the present study were obtained under a high challenge situation, on a study design sufficiently powered using eight animals per group, whereas other efficacy studies *versus* Neotropical sand flies had three or four animals per group [24, 30]. The level of engorgement observed on the females in the control group considering the four different exposure periods was 95.63% (2669/2791). That means phlebotomine sand flies presented an aggressive feeding behavior, which provided a strong challenge for the product. This value was higher than engorgement results observed in other studies conducted in Brazil with *Lu. longipalpis* like those reported by David et al. [31] with 83% (1737/2094) or other New World’s sand flies such as *Mi. migonei* with 76% (1555/2035) [31] and *Ny. intermedia* (*s.l.*) with 42% [30].

High biting rates of *Lu. longipalpis* in dogs, as observed in this study, are an important component of the vector capacity of this sand fly species and is directly related to its zoonotic ability to transmit *Leishmania infantum* on VL endemic areas [8]. The use of insecticide products in the dog population has been suggested as an important measure that could aid in the prevention and control of VL [30, 32–34]. Here, we demonstrate the anti-feeding effect of a topical combination of fipronil and permethrin (Frontline Tri-Act®) with high protection rates for 28 days. Considering the VL dynamics, our results demonstrate that this product could protect dogs from the bites of *Lu. longipalpis* and so could be integrated into canine leishmaniosis control and prevention programs in the Americas.

## Conclusion

The new combination of fipronil and permethrin demonstrated a significant repellent effect against *Lutzomyia longipalpis* bites as soon as it was applied on the dogs, and its repellent efficacy lasted for 4 weeks, with results greater than 80% for 3 weeks. The results suggest that in VL endemic areas, the regular application of the new combination can contribute to the prevention of canine visceral leishmaniosis.

## Abbreviations

VL: visceral leishmaniosis
CVL: canine visceral leishmaniosis
CL: cutaneous leishmaniosis
pi: post infestation

## References

[1] Gould IT, Perner MS, Santini MS, Saavedra SB, Bezzi GM, Mariana I (2013). Leishmaniasis visceral en la Argentina: Notificación y situación vectorial (2006–2012). Medicina..

[2] Oliveira AM, Vieira CP, Diboc MR, Guirado MM, Rodas LAC, Chiaravalloti-Neto F (2016). Dispersal of *Lutzomyia longipalpis* and expansion of canine and human visceral leishmaniasis in São Paulo State, Brazil. Acta Tropica..

[3] Salomón OD, Basmajdian Y, Fernández MS, Santini MS (2011). *Lutzomyia longipalpis* in Uruguay: the first report and the potential of visceral leishmaniasis transmission. Mem Inst Oswaldo Cruz..

[4] Satragno D, Faral-Tello P, Canneva B, Verger L, Lozano A, Vitale E (2017). Autochthonous outbreak and expansion of canine visceral leishmaniasis, Uruguay. Emerg Infect Dis..

[5] OPS - OMS. 2016. Leishmaniasis. informe epidemiológico de las Américas. OPS Informe Leishmaniasis N°4. Available at www.paho.org/panaftosa/index.php?option=com_content&view=article&id=929:informe-epidemiologico-presenta-la-situacion-de-la-leishmaniasis-en-las-americas-en-2012&Itemid=504. Accessed 23 Dec 2016.

[6] Cutolo AA, Camargo DA, Von Zuben CJ (2009). Novos registros de *Lutzomyia longipalpis* (Lutz & Neiva, 1912) (Diptera: Psychodidae) na região Centro-Leste do estado de São Paulo, Brasil. Rev Bras Parasitol Vet..

[7] Dos Santos BK, Cutolo AA, Motoie G, da Silva Meira-Strejevitch CS, Pereira-Chioccola VL (2018). Molecular detection of *Leishmania* (*Leishmania*) *infantum* in phlebotomine sand flies from a visceral leishmaniasis endemic area in northwestern of São Paulo State, Brazil. Acta Trop..

[8] Galvis-Ovallos F, Casanova C, Sevá AP, Galati EAB (2017). Ecological parameters of the (S)-9-methylgermacrene-B population of the *Lutzomyia longipalpis* complex in a visceral leishmaniasis area in São Paulo State, Brazil. Parasit Vectors..

[9] Galvis-Ovallos F, da Silva MD, Bispo GB, de Oliveira AG, Neto JR, Malafronte RD, Galati EA (2017). Canine visceral leishmaniasis in the metropolitan area of São Paulo: *Pintomyia fischeri* as potential vector of *Leishmania infantum*. Parasite..

[10] Otranto D, Dantas-Torres F (2013). The prevention of canine leishmaniasis and its impact on public health. Trends Parasitol..

[11] Brianti E, Gaglio G, Napoli E, Falsone L, Prudente C, Solari Basano F (2014). Efficacy of a slow-release imidacloprid (10%)/flumethrin (4.5%) collar for the prevention of canine leishmaniosis. Parasit Vectors..

[12] Dumont P, Fankhauser B, Bouhsira E, Lienard E, Jacquiet P, Beugnet F, Franc M (2015). Repellent and insecticidal efficacy of a new combination of fipronil and permethrin against the main vector of canine leishmaniosis in Europe (*Phlebotomus perniciosus*). Parasit Vectors..

[13] Miró G, Gálvez R, Mateo M, Montoya A, Descalzo MA, Molina R (2007). Evaluation of the efficacy of a topically administered combination of imidacloprid and permethrin against *Phlebotomus perniciosus* in dog. Vet Parasitol..

[14] Molina R, Espinosa-Góngora C, Gálvez R, Montoya A, Descalzo MA, Jiménez MI (2012). Efficacy of 65% permethrin applied to dogs as a spot-on against *Phlebotomus perniciosus*. Vet Parasitol.

[15] Dumont P, Chester TS, Gale B, Soll M, Fourie JJ, Beugnet F (2015). Acaricidal efficacy of new combination of fipronil and permethrin against *Ixodes ricinus* and *Rhipicephalus sanguineus* ticks. Parasit Vectors..

[16] Dumont P, Fourie JJ, Soll M, Beugnet F (2015). Repellency, prevention of attachment and acaricidal efficacy of a new combination of fipronil and permethrin against the main vector of canine babesiosis in Europe, *Dermacentor reticulatus* ticks. Parasit Vectors.

[17] Fankhauser B, Dumont P, Halos L, Hunter JS, Kunkle B, Everett WR (2015). Efficacy of a new combination of fipronil and permethrin against *Ctenocephalides felis* flea infestation in dogs. Parasit Vectors..

[18] Fankhauser B, Dumont P, Hunter JS, McCall JW, Kaufmann C, Mathis A (2015). Repellent and insecticidal efficacy of a new combination of fipronil and permethrin against three mosquito species (*Aedes albopictus*, *Aedes aegypti* and *Culex pipiens*) on dogs. Parasit Vectors..

[19] Fankhauser B, Irwin JP, Stone ML, Chester ST, Soll MD (2015). Repellent and insecticidal efficacy of a new combination of fipronil and permethrin against stableflies (*Stomoxys calcitrans*). Parasit Vectors..

[20] Beugnet F, Soll M, Bouhsira E, Franc M (2015). Sustained speed of kill and repellency of a novel combination of fipronil and permethrin against *Ctenocephalides canis* flea infestations in dogs. Parasit Vectors..

[21] EMEA, VICH Topic GL9 (GCP) (2000). Guideline on Good Clinical Practices, The European Agency for the Evaluation of Medicinal Products.

[22] Stokes ME, Davis CS, Koch GG. Categorical Data Analysis Using SAS®, 3^rd^ Edition: SAS Press. p. 181–2.

[23] Xiong G, Jin C, Cheng X, Su Z, Hong Y (1994). Deltamethrin bath of domestic dog in the prevention of sandfly bite. Bull Endem Dis..

[24] Molina R, Lohse JM, Nieto J (2001). Evaluation of a topical solution containing 65% permethrin against the sandfly (*Phlebotomus perniciosus*) in dogs. Vet Ther..

[25] Mercier P, Jasmin P, Sanquer A (2003). Prevention of sand fly attack by topical application of a permethrin/pyriproxyfen combination on dogs. Vet Ther..

[26] Otranto D, Paradies P, Lia RP, Latrofa MS, Testini G, Cantacessi C (2007). Efficacy of a combination of 10% imidacloprid/50% permethrin for the prevention of leishmaniasis in kennelled dogs in an endemic area. Vet Parasitol..

[27] Otranto D, Wall R (2008). New strategies for the control of arthropod vectors of disease in dogs and cats. Med Vet Entomol..

[28] Killick-Kendrick R, Killick-Kendrick M, Focheux C, Dereure J, Puech MP, Cadiergues MC (1997). Protection of dogs from bites of phlebotomine sand flies by deltamethrin collars for control of canine leishmaniasis. Med Vet Entomol..

[29] Halbig P, Hodjati MH, Mazloumi-Gavgani AS, Mohite H, Davies CR (2000). Further evidence that deltamethrin-impregnated collars protect domestic dogs from sandfly bites. Med Vet Entomol..

[30] Reithinger R, Teodoro U, Davies CR (2001). Topical insecticide treatments to protect dogs from sand fly vectors of leishmaniasis. Emerg Infect Dis..

[31] David JR, Stamm LM, Bezerra HS, Souza RN, Killick-Kendrick R, Lima JW (2001). Deltamethrin-impregnated dog collars have a potent anti-feeding and insecticidal effect on *Lutzomyia longipalpis* and *Lutzomyia migonei*. Mem Inst Oswaldo Cruz..

[32] Courtenay O, Kovacic V, Gomes PA, Garcez LM, Quinnell RJ (2009). A long-lasting topical deltamethrin treatment to protect dogs against visceral leishmaniasis. Med Vet Entomol..

[33] Sevá AP, Galvis-Ovallos F, Amaku M, Carrillo E, Moreno J, Galati EA (2016). Correction: Canine-based strategies for prevention and control of visceral leishmaniasis in Brazil. PLoS One..

[34] Foglia Manzillo V, Oliva G, Pagano A, Manna L, Maroli M, Gradoni L (2006). Deltamethrin-impregnated collars for the control of canine leishmaniasis: evaluation of the protective effect and influence on the clinical outcome of *Leishmania* infection in kennelled stray dogs. Vet Parasitol..

